# Programmed Sports Therapy (PST) in People with Haemophilia (PwH) “Sports Therapy Model for Rare Diseases”

**DOI:** 10.1186/s13023-018-0777-7

**Published:** 2018-03-05

**Authors:** Thomas Hilberg

**Affiliations:** Department of Sports Medicine, University of Wuppertal, Pauluskirchstr. 7, D-42285 Wuppertal, Germany

**Keywords:** Haemophilia, Rare diseases, Physiotherapy, Sport, Exercise, Rehabilitation, Prevention, Strength, Endurance, Coordination

## Abstract

Sports and exercise therapy becomes more and more integrated in the treatment plan of different diseases. Although the benefits of this therapy are of high quality evidence, e.g. in cardiovascular diseases, no concepts of sports therapy are available as a treatment option for rare diseases.

During the last eighteen years, we analyzed the situation as well as necessity, and developed a model, contents and the concept of the “Programmed Sports Therapy (PST)” for the treatment of PwH (people with haemophilia) as our model of rare disease. Many studies have shown that motoric skills are depressed in PwH, and that this gap to healthy people increases during age. The only way to reduce this progression is an appropriate therapy, adapted to the necessities of PwH. In haemophilia, in particular, physio- and sports therapy treatments should go hand in hand, the first in the acute phase after bleeding, the second later, after the acute phase has finished. One model, which considers all the different challenges, can be the cogwheel model presented here. Since haemophilia is a rare disease, new training concepts are necessary because classical group therapies are often impossible. PST based on the combination of sports therapy camps together with a supervised autonomous home training helps to directly bring the training to the trainee, in order to enhance key competences and improve the individual situation in PwH, and perhaps in patients with other rare diseases.

The experience and scientific data substantiate the success of “Programmed Sports Therapy (PST)” and even this can be a model for other rare diseases.

## Background

This is a narrative review about the necessity and development of a sport therapy concept focusing on rare diseases, and in this special case of haemophilia. This disease requires a multidisciplinary treatment, because hematological, internal as well as orthopedic and muscle skeletal issues are present in people with haemophilia (PwH). Only some decades ago, it seemed to be impossible for PwH to participate regularly in physical exercise. From the perspective of a medical staff person, it is more than understandable that an advice of inactivity had been given as a rule, because factor concentrates to treat possible bleeding events were still lacking. Due to an increasing opportunity of sufficient treatment options, there was and still is a change of thinking. This is helpful in the disease management of PwH today [1]; on the one hand to treat haemophilia specific, but also disease unspecific issues, on the other hand. More and more PwH participate in a variety of sports [2] and various, often non-randomized studies describe the benefits in different fields, however on a low level of evidence [3]. In the last decades it has become gradually clear that physical exercise is more than exercise only. In a greater degree, it is an approved therapy, in the hands of experienced sports therapists and adapted to the needs of patients, even with rare diseases. This narrative review describes the necessity, possibilities, the success and a way of incorporating sports therapy in the treatment of PwH as a model of rare disease, based on our and the important research of many other colleagues (literature research, main emphasis PubMed and Google Scholar) during the last eighteen years, with our start in the end of the 90^ies^.

### Physical performance in PwH

#### Basic motoric skills in PwH

From the sports medicine perspective, we distinguish between five different basic motoric skills: strength, coordination, endurance, flexibility and speed. Except for the last one, all these elements are of main importance in respect of physical health, and partly also psychological health. Whereas strength and coordination, together with flexibility, have a main role in inter- and intraarticular joint play, endurance has a further overall physical effect, not least, because it improves cardiovascular [4, 5], metabolic [6], as well as immunological function [7]. These important motoric skills for every human being are restricted in PwH, if bleedings were present in the history of the patients, but sometimes also in patients without reminded bleedings. In this case, this could be due to unknown silent bleedings and/or inactivity.

#### Muscle strength in PwH

In an older study, we could show, that muscle strength is attenuated in the lower limp of adult PwH [8], whereas Falk et al. had published the same findings in children with haemophilia [9]. This is of main importance because the *M. quadriceps* is a main shock absorber for the knee as well as the hip joint. In their cross sectional study, Baker et al. revealed that quadriceps muscle weakness has a close relationship to knee osteoarthritis in nearly all compartments [10]. The quadrizeps together with sufficient ischiocrural muscles strength are mainly responsible for a solid joint play of the knee. Brunner et al. could show that there exists a difference in quadriceps strength of approximately 30% between adult subjects with or without haemophilia [11]. This difference becomes more prominent in the older age, e.g. 40–49 years - 35-36%; 50–70 years - 53-61%, but is also present in the younger groups (18–29 years - 10-13%; 30–39 years - 20-23%). The definite reason for the widening of this gap is not clear; it still is a mixture of progression of arthropathy as well as reduced utilization of the muscles. Although the extension of the impact of muscle weakness on the development of haemophilic arthropathy has not been clearly investigated yet, the data from osteoarthritis studies support the hypothesis, that muscle weakness lead to a faster progression in destructive joint disease and therefore should be avoided in PwH, as good as possible. Beside the destructive mechanisms induced by blood components such as iron as well as inflammatory cytokines in the joint [12, 13], the necessary immobilization after joint bleeding indeed includes negative consequences for the affected muscles, particularly if the time space of immobilization is too long.

#### Implication of long-lasting immobilization or disuse

The effects of disuse have been comprehensively investigated by de Boer et al. [14, 15] and Narici et al. [16]. The authors used a unilateral lower-limb suspension model in young healthy men, where the *“dominant leg was kept in slightly flexed position by use of straps, suspending the foot of the dominant leg above the ground while walking with crutches”* for a period of 14 and/or 23 days. The torque of the knee extensor was reduced by 15% after 14, and 21% after 23 days [14]. In addition to the functional loss, the suspension also resulted in a distinct reduction in the muscle anatomical cross sectional area by approximately 5% after 14 and 10% after 23 days [14]. Investigations of the metabolic muscle situation after disuse show a reduction in myofibrillar protein synthesis, tendon collagen synthesis accompanied by a decreased phosphorylation of FAK (focal adhesion kinase), a cell signalling molecule, which is associated with mechanotransduction. The PKB-P70-mTOR protein anabolic pathway, however, remained unaffected [15]. The last one is probably downregulated in animal models during disuse atrophy [17]. Sufficient resistance training after this suspension is able to compensate a lot of these disuse effects as shown by Brocca et al. [18]. Therefore, the main problem in PwH is not a single necessary immobilization over a short but sufficient time period, but an insufficient rehabilitation and false immobilization afterwards and/or repeated bleeding with recurrent periods of immobilization. That is why immobilization should be adapted to the needs and is not equal to absolute disuse. Whereas weight-bearing to the vulnerable joint after bleeding counteracted the rehabilitation process, isometric exercises and/or CPM (continuous passive motion) or CAM (continuous active motion) devices can be very useful in this early process of rehabilitation. The consequences of weight-bearing on cartilage during the vulnerable phase after joint bleeding have been addressed in animal models by Hooiveld et al. [19] and Ravanbod et al. [20]. On the other hand, consequences of muscle atrophy could be shown by Brunner et al. [11]. Muscle weakness in one limp is associated with higher risk of inter-extremity difference (IED), and this asymmetry between the two limps, distinctly increases during aging [11]. In addition, this IED of strength is based on a protection of the more affected limp but de facto often goes along with an overload of the contralateral limp [21]. This explains the clinical picture of contralateral more affected joints in many PwH, e.g. knee left, ankle right or otherwise. Therefore, physio- and sports therapy should focus on IED to guarantee a balanced weight load. In the other case, the process of muscle weakness and IED is accompanied by an increased fluctuation as shown by Gonzales et al. [22] and Brunner et al. [23], which results in lower steadiness during voluntary contraction. The consequences of this in PwH should be addressed by future studies. However, Carville et al. [24] underpinned that elderly, who tend to fall, show a lower isometric contraction steadiness of the quadriceps muscle, compared to young subjects as well as elderly, without the tendency to fall. Not least, also the neuromuscular innervation is influenced by processes in the development of haemophilic arthropathy.

#### Neuromuscular control and coordination in PwH

EMG studies initiated by Kurz et al. confirm that neuromuscular innervation measured by surface EMG is influenced in parts in relation to the extent of haemophilic arthropathy in PwH [25–27]. These processes influence inter- and intramuscular coordination, too [21]. The fact that coordination is decreased in PwH has been investigated, for instance, in 2001 [8] as well as by Gallach et al. [28] by different methods. Interestingly, Gallach et al. demonstrated that PwH without haemophilic arthropathy also had a worse balance unlike controls in a posturographic analysis [28]. Therefore, the rehabilitation process should not only focus on the muscle atrophy after bleeding, but also on the potentiation of disuse and aging processes. This is due to the fact that aging alone can lead to the process of sarcopenia in older ages. Since PwH become older especially in the industrial world, this will be of more and more importance now and in the near future [29, 30]. In many studies it could be seen that flexibility, as another relevant factor is often reduced, particularly in adult PwH [8] but also in children with haemophilia [31].

#### Flexibility and endurance in PwH

Flexibility is not only necessary for normal daily activities but also a protection for the joint chain and the joint play. Reduced flexibility leads to a higher stress, not only for the affected, but also the adjacent joints. At least, endurance is also influenced by presence of haemophilia. This is not relevant to every PwH, but if immobilization had been necessary previously or physical inactivity is still present, it influences endurance performance as well. In an earlier study we could depict that maximal as well as submaximal endurance performance was depressed in a group of young to middle aged (range 16–44, mean 32 years) PwH [32], whereas data from Falk et al. confirm the same in children with haemophilia [9]. Since life expectancy in PwH has increased due to better treatment options, endurance capacity becomes more important, too. Endurance capacity is closely related to the prevention of metabolic as well as cardiovascular-related mortality risk. A clear reduction in total, as well as in cardiovascular-related mortality risk due to primary prevention by physical exercise, has been described in people without haemophilia [5]. Data in PwH are still lacking. Although these deficits of motoric skills are clearly present in adult PwH, Seuser et al. could show in a comprehensive study that also in children with haemophilia some deficits are present. The authors studied sport-specific motor performance in 285 PwH between 8 and 25 years and underpinned lower abilities in the one leg-stand test, the mobility of the lower extremity, the strength ratio of chest and back muscles as well as in the endurance test in comparison to a control group without haemophilia. However, some parts of the results were better in the PwH group than in healthy peers [33].

In conclusion, all the data mentioned above confirm that changes in the motoric skills still not only exist in adult PwH, which are in these cases very often directly visible, but also in children with haemophilia, where the diagnostic needs to be more sensitive. Therefore, for a higher sensitivity, other diagnostic tools such as gate or motion analysis seemed to be helpful [31, 34]. For both groups, adults and children, sports therapy will be of advantage.

### Physical exercise in PwH

Although non-therapeutic physical exercise and sports are not the main topic of this review, this part should be also briefly addressed. Regarding this topic, excellent reviews exist, e.g. by Gomis et al. [1], Mulder et al. [35], Jones et al. [36] and Strike et al. [37]. The last one is an important current systematic review that shows that only a small number of sufficient studies exist. However, they describe *“most exercise interventions produced improvement in one or more of the measured outcomes including pain, range of motion, strength and walking tolerance. Hydrotherapy may be more effective than land exercises for pain relief in adults. Functional exercises such as treadmill walking and partial weight bearing exercises seem to be more effective than static or short arc exercises for improving muscle strength”*. It should be pointed out that the advice for an appropriate kind of sports should be given by experts working in the haemostaseological and sports medicine/orthopedic field together with physio- and/or sports therapists. The first specialist has to optimize possible factor treatment; the second one proves the conditions and collectively they will find the right type of sports. In many cases, this will be an individual counselling on the basis of the individual conditions of the PwH and type of sports they desire. It is clear that as much as components, such as contacts inside sports, possible trauma as well as eccentric stress play a role in the selected type of sports, this will not be ideal for PwH. In some sports, the judgement whether it is a “red flag” or not is very easy, e.g. in boxing or rugby. This does not apply to every kind of sports, though. For instance, karate seems to be the wrong type of sports but practicing “kata” only, which includes specialized coordination training without any physical contact, would be feasible for PwH also. So, the judgement should not only address sports in toto, but should also focus on possible parts of sports, which are relevant. This makes the recommendation more complex and should be done by an experienced team, which survey both medical specializations. This team has also to consider the fact that children with hemophilia will grow up and that the impact and the performance requirements within the particular type of sports, will also increase. It is probably better to help finding an ideal type of sports in the younger age already, than to stop the subject practicing a non-ideal type of sports due to injuries or high impact performance requirements. This is likely to result in frustration and/or quite often in complete refusal of physical activity. Different sports classifications for PwH exist, based on the points mention above. Some of them categorize the kind of sports symbolized by a traffic light, whereby red and green are often non-debatable, whereas yellow, should be further discussed based on the individual conditions [36].

Summarizing the data above, sports and exercise is important, but it is more than fun and motion. Targeted therapeutic goals in a controlled situation of therapy is medicine, but this will be explained in the next section.

### Sports therapy

#### Background of sports therapy

**Fig. 1:**
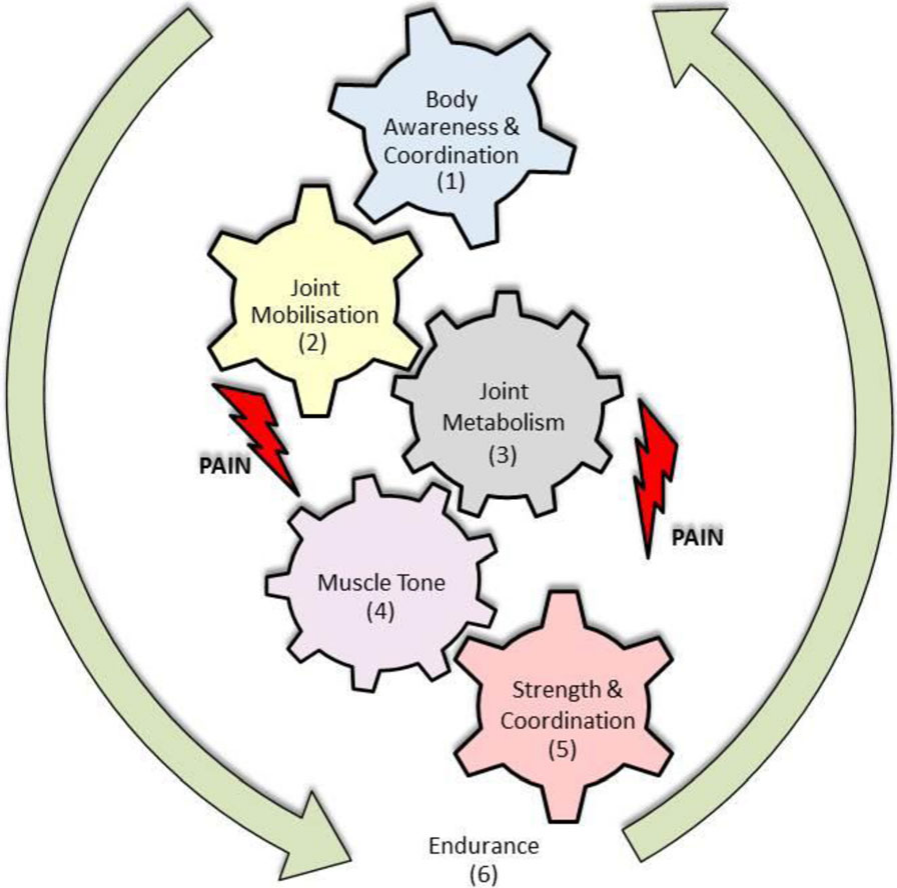
Cogwheel model of Sports Therapy; Number (1–6) see also Fig. 2

Since the term of “Sports Therapy” is not common in every country and sometimes ambiguous in use, it is important to firstly present a definition: *“Sports therapy is an exercise therapy - based on the motoric skills - which is prescribed by a physician including behavior-orientated components, planned and dosed by sports therapists, but controlled together with physicians and performed - supervised by therapists - by the patient alone or in a group”* (adapted from Hilberg in Astermark et al. [38]). Sports therapy doesn’t stand in contrast to physiotherapy; it rather works hand in hand together in this field of work, helping to amplify or potentiate physiotherapeutic effects. Whereas physiotherapy is a medical necessity in the acute phase after bleedings, sports therapy is to take into consideration after the acute phase of bleeding has ended. Especially, but not only, in regions where physiotherapists are not nearby, sports therapy is helpful for PwH to improve the situation and the mobility in the post-acute or chronic phase, whether e.g. weight-bearing is possible again. For the therapeutic approach, we have developed a model with central elements focused by sports therapy. The cogwheel model as a central approach is shown in Fig. 1 (adapted from Hilberg in Astermark et al. [38]). This model comprises six segments which individually count on their own, but are also working together. The first one is “body awareness” in combination with “coordination” to better kinesthesia and reducing false movements as a basis for training the other components. Improving “joint mobilization” and activation of the “joint metabolism” are important steps, which can be trained by a well instructed and supervised PwH, also by himself alone. Regulation of “muscle tone” is important, because Kurz et al. described an increased “muscle tone” in knee extensor muscles in PwH with affected joints [26]. An enhancement of “muscle strength and coordination” should be additional central steps followed or accompanied by an “endurance training” in general.

#### Pain and sports therapy

Pain, especially present in PwH with affected joints, hinders the therapy success and should be addressed by a sufficient therapy [39]. Bank et al. substantiate in their excellent review the negative consequences of limb pain for muscle activity, strength and proprioception [40]. The changed redistribution of activity within and between muscles leads to an altered distribution of load, movement and variability as shown by Hodges et al. [41] and with it increases the impact on joints with consequences for the cogwheel model.

#### Beneficial effects of sports therapy

Following this model, different studies have shown that this therapy is successful. In an early cohort study - published in 2003 - we were able to demonstrate that muscle strength in PwH can be improved by sports therapy although patients are limited by haemophilic arthropathy [42]. This study result is in accordance with other studies of different research groups and also in line with us, that a sufficient training seemed to be able to improve muscular strength also in patients in different ages and with or without obvious haemophilic arthropathy [43–46]. However, most of these studies were cohort studies/case reports or studies in children and criticized, due to the fact, that this kind of studies has almost always the possible bias that the active group is more motivated, which can possibly influence the study results. For handling this critic, we initiated a randomized controlled study which has been published just recently [47]. In this study, it could be shown that the results of the cohort studies could be clearly confirmed in adult PwH also at the highest possible study design. It is important to point out the necessity of a well-trained therapist, which has the ability to teach, control and supervise the sports therapy training. A combination of strength with coordination training is relevant and the study results demonstrate the feasibility and improvement [47]. The major importance of strength and coordination in the context of prevention of falling is addressed by Forsyth et al. [48], and falling and fall risks become more and more a topic in PwH [49]. Although some studies indicate changes in flexibility, this is directly dependent on the situation of the affected joint. The outcome will be less, whether contraction is fixed, so that the best range of motion (ROM) should be maintained as good as possible. Blamey et al. have also claimed to address any ROM limitations after a bleeding episode has resolved [50]. Sports therapy as well as physiotherapy are often helpful, but if conservative treatments fail, other treatments such as radiosynoviorthesis or joint surgery are unavoidable [51]. However, also in the case of e.g. total knee arthroplasty the clinical outcome is influenced by the preconditions, because the outcome is lowered, e.g. in PwH, with stiff knees [52] and hospitalization is prolonged. At least, it could also be underpinned that endurance is also enhanced after sports therapy treatment. In this direct context, just for once the musculoskeletal system doesn’t stand in the center of interest. Especially in the industrial world, PwH have the same life expectancy as people without haemophilia and, therefore, also other diseases such as metabolic and cardiovascular diseases will be more present in PwH. The reduction of metabolic and cardiovascular diseases by the improvement of endurance performance is confirmed by sufficient studies. In addition, we know that physical endurance exercise can modulate the immunological function, but it is still unclear if this is also helpful in the context of HIV and/or Hepatitis infection, which are present in some PwH. Today it is undoubted that sports therapy is helpful to reduce deficits in the motoric skills in PwH. This could be shown by measuring objective as well as subjective physical performance parameters. The latter are not unimportant because the subjective physical performance is quite different to the objective physical performance in many cases and sometimes give a better overview of the personal reflexion concerning individual physical performance [53]. This should be taken into consideration in respect of measuring physical performance. Not only qualities of performance are influenced by sports therapy, but also the quality of life in parts could be improved by a sports therapy program, as Runkel et al. revealed, too [54]. Many therapists agree that advising physical activity is not enough, now it is “time to prescribe” as Lobet et al. title their publication [55]. However, one central question is how to train PwH. In cardiovascular diseases with a higher prevalence it seemed to be easier because sports therapy groups or similar therapeutic options are more common in every bigger city.

Sports therapy is helpful for PwH. However with a low prevalence, such as in Haemophilia and other rare diseases, these options are often locally not present, which necessarily leads to the development of other concepts. On the basis of these backgrounds, the concept of *“Programmed Sports Therapy”* has been developed during the last years.

### Programmed sports therapy

**Fig. 2:**
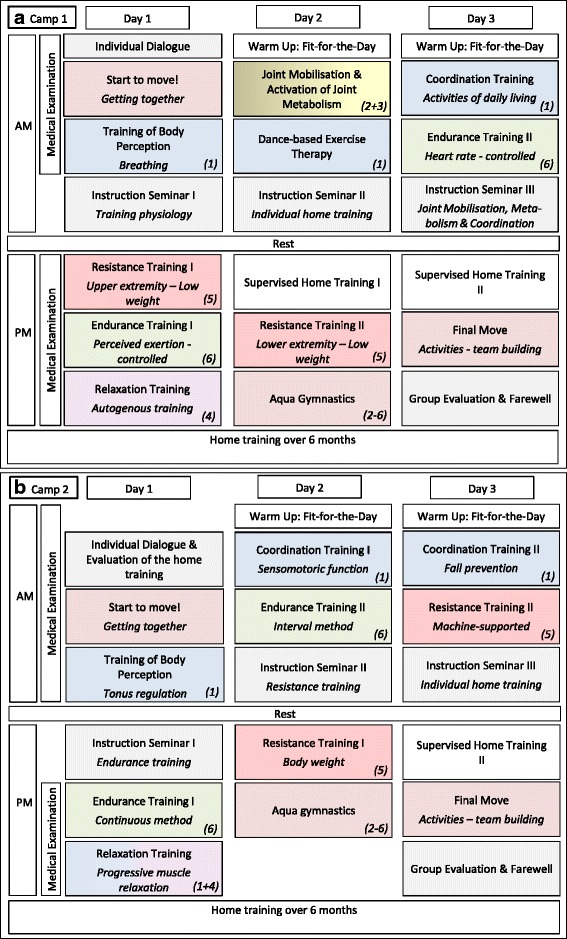
**a** + **b**: Exemplary one-year course – Sports Therapy Camps and Home Training. Number (1–6) see Fig. 1 - Cogwheel Model; every practical training session includes approx. 45–60 min, depending on intensity; theoretical training session approx. 60 min

The background of the *“Programmed Sports Therapy”* is to develop key competences of sports therapy in PwH, to enable PwH more and more, step by step to manage their own training, supported and supervised by an experienced team. This could be a model also in other rare diseases. The concept can close the gap between PwH and treatment centers, in some cases over huge distances. This gap is a central problem also in other rare diseases. Therefore, this concept combines sports therapy camps with individual and group training in typical group settings with a supervised autonomous training at home or in training centers nearby. The sports therapy camps include theoretical as well as practical training to enhance the individual competence in the field of sport science, medicine as well as the specific hemostatic disorders. An exemplary one-year course, which includes two consecutive sports therapy camps together with home training, is shown in Fig. 2. This example could be adapted to fit the needs of other rare diseases. The components and the background are based on the cogwheel model, which is mentioned in Fig. 1. The numbers are corresponding in both figures and concrete exercises for every component are available on http://www.haemophilia-exercise.de; otherwise this would be going beyond the intension of this publication. In order to reach the specific training targets in PwH, the different exercises have to be adapted to the individual situation of PwH, e.g. by type, used position, possible ROM, frequency, duration as well as intensity of exercise considering age, joint status, further diseases, as well as the availability of clotting factors. For reducing weight bearing of the joints, water as a medium can be used for sports therapy, which is implicitly helpful. The detailed training targets and adaptations differ from training programs for healthy persons, whereas the general goals are similar, e.g. improving quality of life. The concept followed physiological training rules, and is based on the theoretical and practical experience over years. With this concept, it is possible to directly bring the training to the trainee and not the other way round. This avoids long journeys to the training center, which is also helpful in other rare diseases. The sports therapy camps should be initiated 2–3 times per year, such as in our case for 3–4 days, to guarantee sufficient time for education and training. During the last decades, we developed and adapted lots of different exercises to the needs of our PwH. All these different exercises can be consulted on the homepage mentioned above. After the necessary medical examination at the start, it is important to choose exercises with an adapted power and execution, which possibly implies the specification of execution concerning speed, position, angle and others. The follow up of the camps should guarantee that the competence in this field will increase in PWH, step by step over time, and corrections of motions sequences will be made to avoid the development of motion errors. During this long-time period of eighteen years and after more than 35 initiated sports therapy camps in Germany and Switzerland, we could certainly underpin that these camps are possible to realize, without any complications and that they are helpful in the development of personal competence of PwH, in this field. Between the sports therapy camps, the PwH train autonomously and supervised by training-protocols prepared by the sports therapist, which can reduce costs, because these are also relevant in the treatment of PwH [56]. Thus, this concept of *“Programmed Sports Therapy”* can be the key to include sports therapy to a comprehensive therapy regime in PwH, and adapted, as well as other rare diseases, if physical training is possible.

## Conclusion

During the last eighteen years, sports therapy has been developed in the treatment of PwH and even should be developed for other rare diseases. Since PwH often suffer from impaired motoric skills due to bleedings as a result of the haemostatic disorder, sports therapy is significantly helpful to better the reduced function. One potential therapy concept is based on the cogwheel model presented here. Since haemophilia is a rare disease, other concepts than classical group therapy are necessary. The concept of *“Programmed Sports Therapy (PST)”* focusses on present necessities and includes the aim to enhance competences in the use of sports therapy in PwH. On a final note, PST can also be helpful in other rare diseases.

## Abbreviations

CAM: Continuous active motion
CPM: Continuous passive motion
e.g.: For example
EMG: Electromyogram
FAK: Focal adhesion kinase
HIV: Human immuno-deficiency virus
IED: Inter-extremity difference
mTor: Mammalian target of rapamycin
PKB: Protein kinase B
PST: Programmed Sports Therapy
PwH: People with haemophilia
ROM: Range of motion
